# Optoelectronic system for brain neuronal network stimulation

**DOI:** 10.1371/journal.pone.0198396

**Published:** 2018-06-01

**Authors:** Mikhail A. Mishchenko, Svetlana A. Gerasimova, Albina V. Lebedeva, Lyubov S. Lepekhina, Alexander N. Pisarchik, Victor B. Kazantsev

**Affiliations:** 1 National Research Lobachevsky State University of Nizhny Novgorod, Nizhny Novgorod, Russia; 2 Center for Biomedical Technology, Technical University of Madrid, Campus Montegancedo, Pozuelo de Alarcón, Madrid, Spain; Nanjing University, CHINA

## Abstract

We propose an optoelectronic system for stimulation of living neurons. The system consists of an electronic circuit based on the FitzHugh–Nagumo model, an optical fiber, and a photoelectrical converter. We used this system for electrical stimulation of hippocampal living neurons in acute hippocampal brain slices (350-μm thick) obtained from a 20–28 days old C57BL/6 mouse or a Wistar rat. The main advantage of our system over other similar stimulators is that it contains an optical fiber for signal transmission instead of metallic wires. The fiber is placed between the electronic circuit and stimulated neurons and provides galvanic isolation from external electrical and magnetic fields. The use of the optical fiber allows avoiding electromagnetic noise and current flows which could affect metallic wires. Furthermore, it gives us the possibility to simulate “synaptic plasticity” by adaptive signal transfer through optical fiber. The proposed optoelectronic system (hybrid neural circuit) provides a very high efficiency in stimulating hippocampus neurons and can be used for restoring brain activity in particular regions or replacing brain parts (neuroprosthetics) damaged due to a trauma or neurodegenerative diseases.

## Introduction

Neuroprosthetics is one of the most promising areas of interdisciplinary research in the field of neuroscience. One of the neuroprosthetics aims is the development of electronic devices implanted into the brain to reestablish its missing or wrong functionality due to injury or disease [1]. In this context, a brain-machine interface has been an object of intensive research in recent years, including neuroprosthetic applications [2–4]. In particular, neurointerfaces were successfully applied for limb prostheses control in patients with spinal cord trauma, where remaining neural pathway signals were used to control prostheses [5,6]. Moreover, neurointerfaces and neuroprostheses are on high demand for restoring visual (artificial retina prostheses) and auditory (cochlear implants) sensory functions [7,8].

The history of neuromorphic technologies began in the late 1980s, and significant advances have been achieved ever since thanks to the progress in electronics [9,10]. Nowadays, the development of neuromorphic technologies is of great importance for neuroprosthetics and humanity. One of the neuromorphic technological solutions is the implementation of electronic devices which mimic the electrophysiological behavior of real neural networks. Neuromorphic technologies are relevant not only to medical applications, but also to non-medical tasks, such as neuromorphic information processing, intellectual adaptive automatic control systems, biorobots, etc.

In the last decades, different electronic circuits have been designed to generate neural dynamics. They are distinguished by their biological relevance, a number of simulated ion channels and a number of represented dynamic modes [11–18]. Collective dynamics of artificial neurons were also extensively studied [19–22] with concentrated efforts in modelling and implementing synaptic connections in hardware. It should be noted that synaptic plasticity is one of the most sophisticated and challenging features of neuronal network dynamics to be implemented in hardware. The synaptic electronic circuits were built to transform presynaptic voltage pulses to postsynaptic currents with some synaptic gains. Different strategies of hardware implementation of synaptic circuits were employed (for review see [23,24]). Recent advances in nanotechnologies allowed miniaturization of artificial synapses by constructing carbon nanostructures which mimic synaptic dynamics [25]. Some circuits [26] also provided spike-timing dependent plasticity (STDP) responsible for long-term memory.

The first optical fiber synaptic sensor (OFSS) was designed in 2011 [27] on the base of an erbium-doped fiber laser which linked two electronic FitzHugh-Nagumo neurons. The functionality of this artificial optical synapse has been tested both theoretically and experimentally in two different configurations: under laser loss modulation and under diode laser pump modulation [28]. These two configurations allowed additional flexibility in the parametric control of this “optical synapse”. The electrical postsynaptic signal was used to synchronize the postsynaptic and presynaptic neural oscillators.

In this work, on the base of the proposed OFSS we construct an optical fiber interface to connect the analog electrical FitzHugh–Nagumo neuron with living neurons. We also study possibilities for stimulating living neurons by an optical signal, transmitted from the electronic presynaptic neuron, and analyze synchronization between the artificial neuron and living neurons.

## Materials and methods

### Ethics statement

All experimental protocols in this research were reviewed and approved by the Bioethics Committee of the National Research Lobachevsky State University of Nizhny Novgorod (protocol no. 10 from 19.07.2017); the experiments were conducted in strict accordance with Act 708n (23.08.2010) of the National Ministry of Public Health of Russian Federation approving the rules of laboratory practice for the care and use of laboratory animals and Council Directive 2010/63EU of the European Parliament (September, 22, 2010) on the protection of animals used for scientific purposes. C57BL6J mice and Wistar rats were anesthetized by isoflurane and sacrificed by cervical vertebra dislocation.

### Hybrid neural circuit

**Fig 1:**
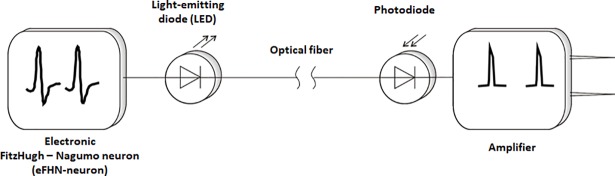
Scheme of the hybrid neural circuit.

**Fig 2:**
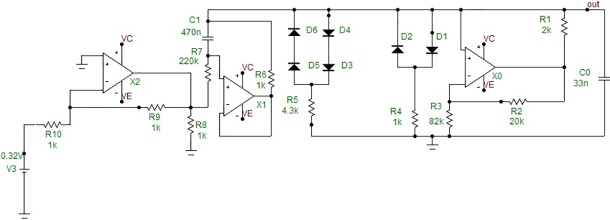
Electronic scheme of analog FitzHugh–Nagumo neuron circuit. Two sets of diodes produce cubic nonlinearity.

The designed hybrid neural circuit consists of an analog electronic FitzHugh–Nagumo (FHN) neuron, a light-emitting diode (LED), an optical fiber, a photodiode, and an amplifier. This neurointerface operates as follows (Fig 1). The electronic neuron based on the FHN circuit [19,28] (Fig 2) modulates the intensity of LED emission. It should be noted that the dynamics of the FHN neuron qualitatively represents the main features of a living neuron, namely, the presence of an excitability threshold, and resting and spiking behaviors. The potentiometer in the circuit provides control over the dynamical modes of the FHN generator, i.e., its resting and spiking states.

**Fig 3:**
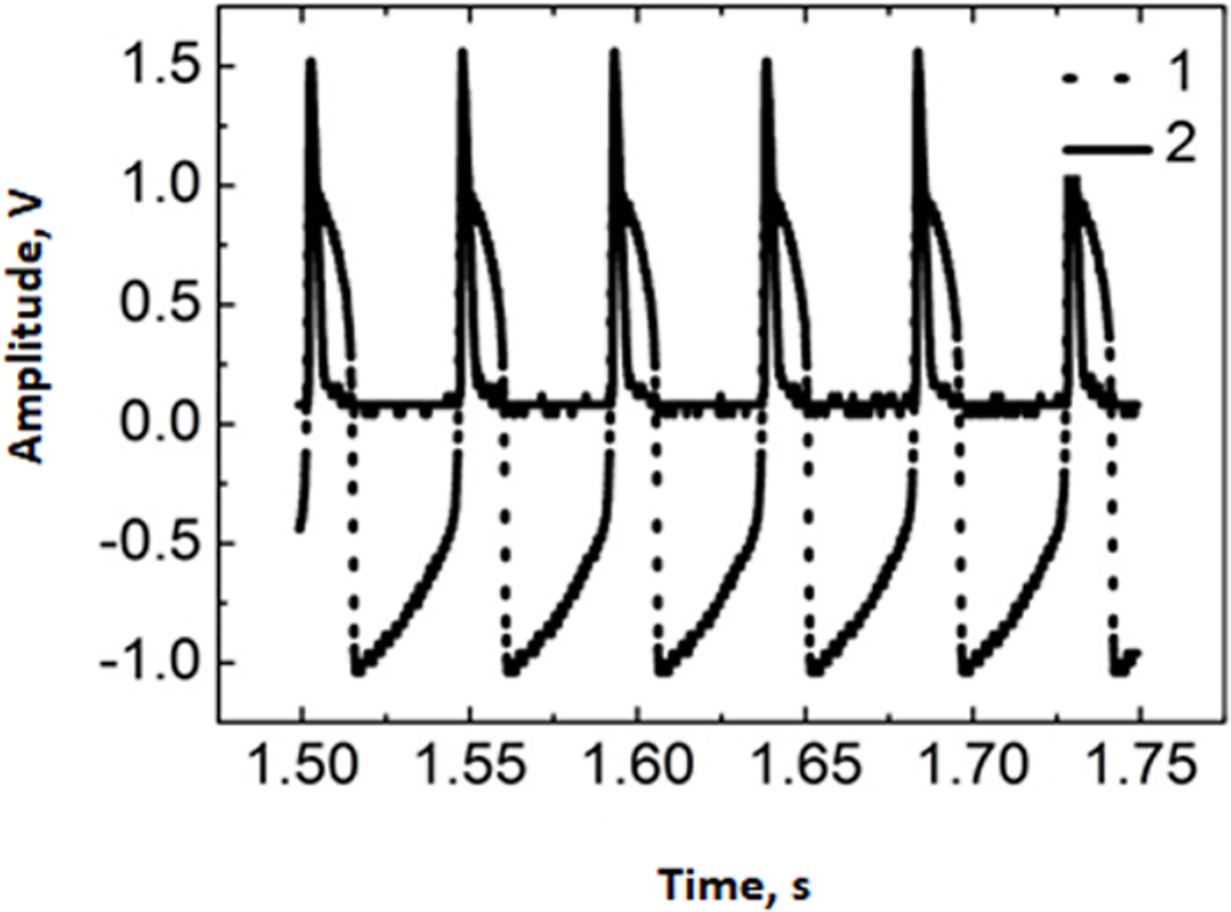
Oscillograms of FHN neuron (curve 1) and amplified photodiode (curve 2) voltages.

The spiking frequency of the FHN neuron is varied in the 12–30 Hz range and the spike duration in the 10–25 ms range. The FHN circuit output signal is registered by an oscilloscope after passing the emitter transistor amplifier. It is worth noting that there is a possibility of reducing the amplifier size to a nanoscale. The spiking amplitude of the FHN neuron output is controlled by a potentiometer in the 0.6–5 V range. The LED light is controlled by the FHN circuit and sent through a multimode optical fiber ending with a photodiode for photo-electric conversion. The resulting electrical signal is amplified for extracellular neural stimulation of a living neuron. The oscillograms of the FHN neuron (1) and amplified photodiode (2) signals are shown in Fig 3. The signal of the photoelectric conversion circuit is used to stimulate living neurons by voltage pulses of 1–10 ms duration and 1–6 V amplitude.

### Biological experiments with hybrid neural circuit

**Fig 4:**
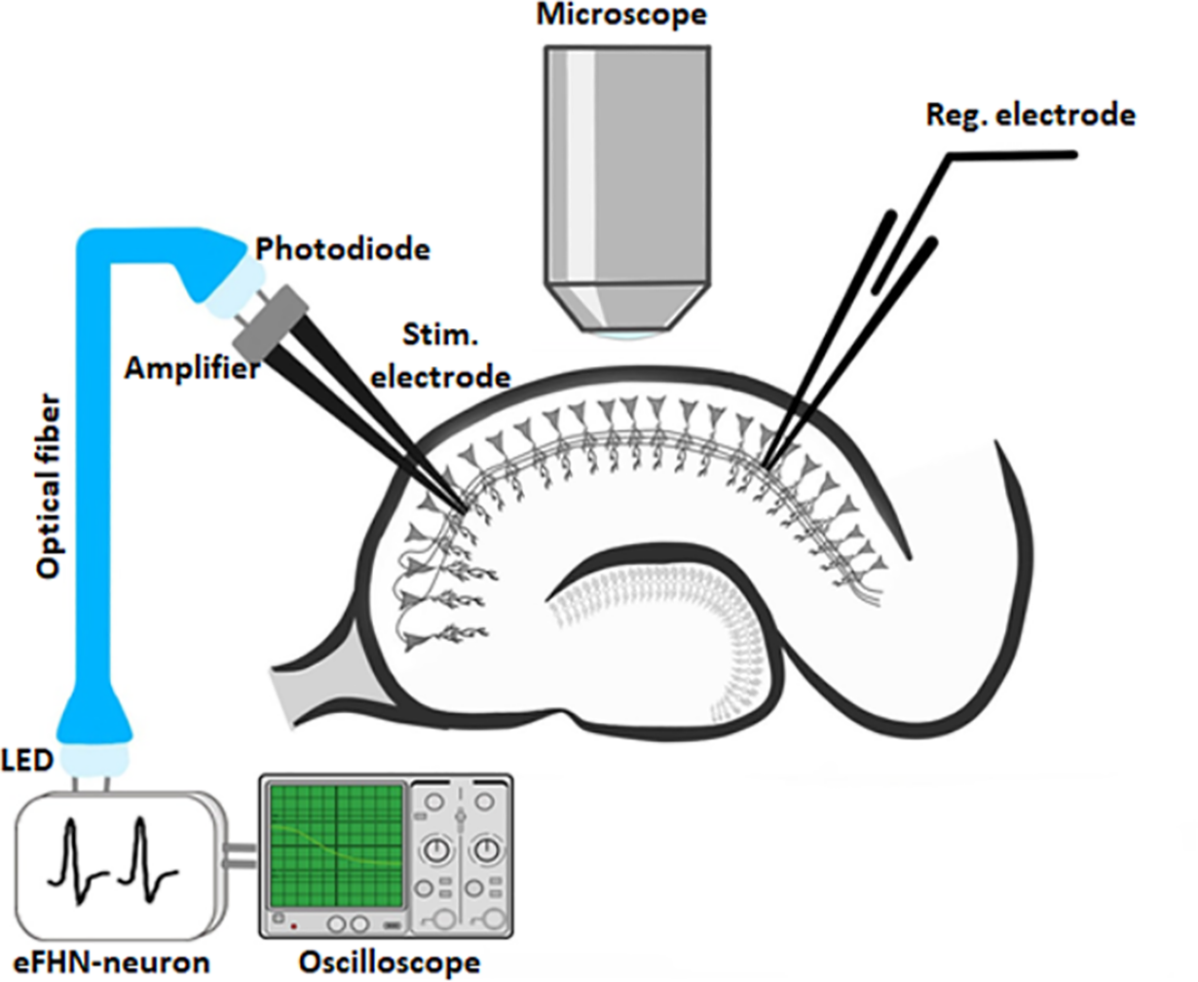
Scheme of our biological experiment with hybrid neural circuit.

We carry out biological experiments with acute hippocampal brain slices (350-**μ**m thick) obtained from a 20–28 days old C57BL/6 mouse and a Wistar rat. The hippocampal slices are incubated for one hour in a modified Ringer solution at 24–34°C, which weakens the cutting effects. The brain slices remain efficient for about six hours. After incubation, the slices are placed in a microscope camera for recording electrophysiological potentials. Due to its unique morphological and functional structure, this biological object (hippocampal slices) makes synaptic transmission and plasticity possible to study. The signal from the electronic neuron generator is transmitted through the optic fiber communication channel to the bipolar electrode (FHC, Bowdoinham, USA) which stimulates Schaffer collaterals (axons of pyramidal neurons in the CA3 field) in the hippocampal slices (Fig 4).

For biopotentials registration from the neuronal cells, we use a glass microelectrode with a 2–5 MOhm resistance, filled with Ringer solution containing 119 mM NaCl; 2.5 mM KCl; 1.3 mM MgSO_4_; 1 mM NaH_2_PO_4_; 26.2 mM NaHCO_3_; 1 mM CaCl_2_; 11 mM D-Glucose (pH 7.4; 290–300 mOsm). The responses from individual neurons and a group of neurons were recorded using patch-clamp and extracellular recording techniques, respectively. The location of the recording and stimulating electrodes in the hippocampal slices is selected to get maximum response.

The recordings are done with the patch-clamp amplifier Multiclamp 700 B (Molecular Devices), filtered at 2.8 kHz and digitized at 5 kHz with the NI PCI-6221 card (National Instruments). The data is visualized and stored with the software WinWCP supplied free of charge to academic users by Dr. John Dempster from the University of Strathclyde, UK.

### Population spikes and excitatory postsynaptic potentials recording

**Fig 5:**
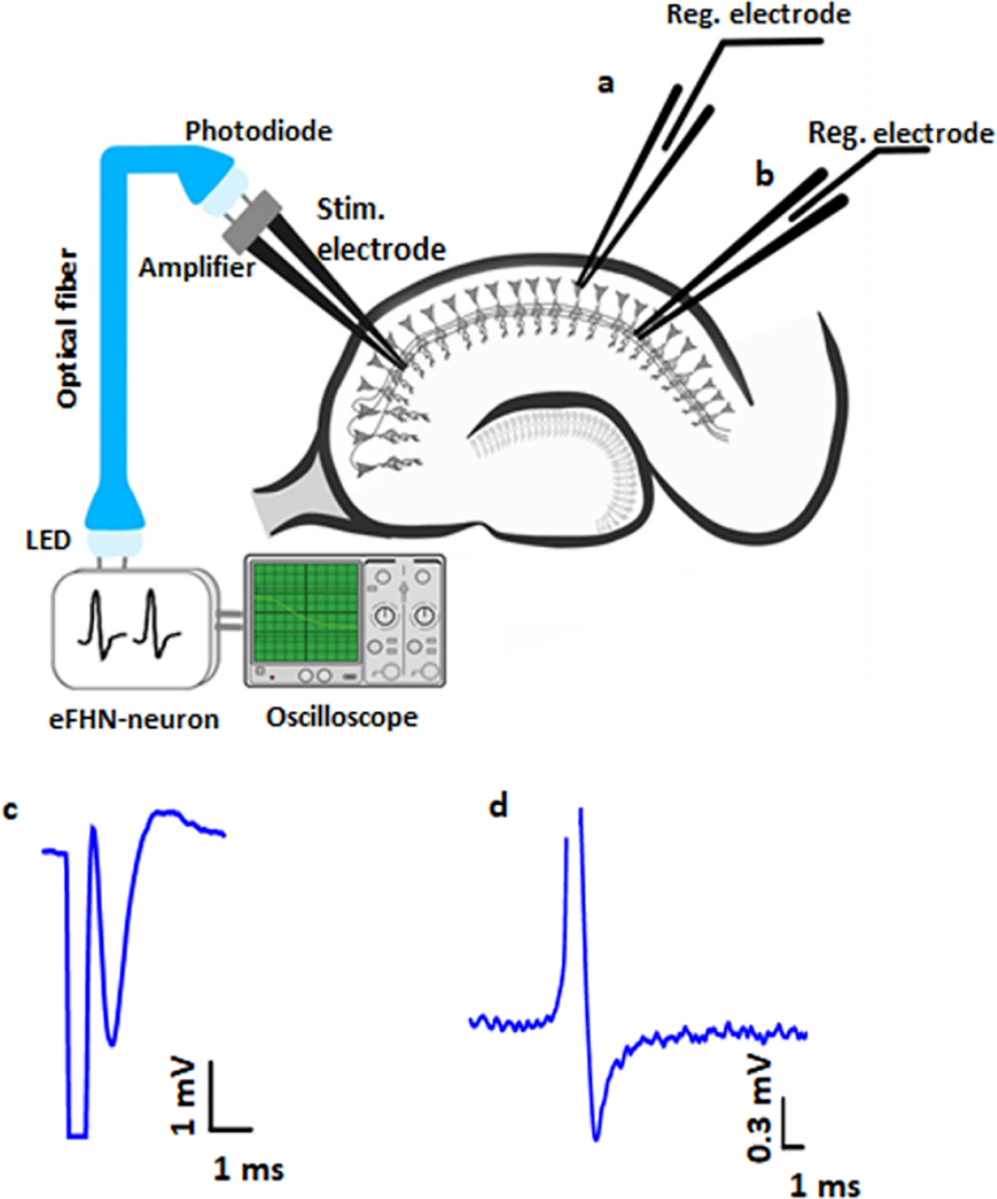
Experimental scheme with electrodes location for recording (a) population spike and (b) excitatory postsynaptic potentials, and (c,d) corresponding neuron responses. Registration electrode detect (a) evoked population spike from pyramidal neurons somas and (b) excitatory postsynaptic potentials from dendrites of pyramidal neurons during electrical stimulation of Shaffer collaterals.

In order to record population spikes, which are the summarized potential of pyramidal neuron somas in the hippocampal CA1 area, we stimulate Schaffer collaterals either by single pulses or a pulse train from the hybrid neural circuit. Simultaneously, the excitatory postsynaptic potential (EPSP), i.e. the total extracellular potential of the CA1 pyramidal neurons, is recorded. The experimental scheme and typical neuron responses are shown in Fig 5.

## Results and discussion

### Synchronization of extracellular neuronal responses by hybrid neural circuit signals

**Fig 6:**
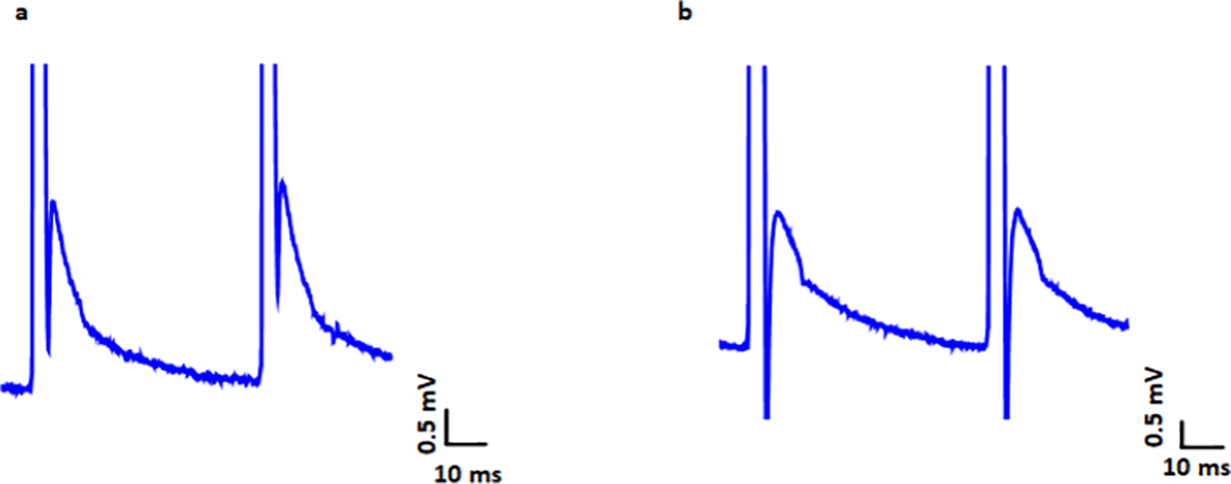
Recording of EPSPs after tonic stimulation from hybrid neural circuit with (a) 12-Hz 1-V pulses and (b) 20-Hz 2.5-V pulses.

We carry out several experiments on neuronal stimulation by the hybrid neural circuit. Typical recorded extracellular potentials after tonic stimulation are shown on Fig 6.

We should note that stimulus artifacts with an amplitude of up to several hundred millivolts are also observed in the recorded signal. These artifacts can easily be distinguished from synaptic responses, because the postsynaptic potentials have a duration of several tens of milliseconds, that is longer than the artifact duration. We observe positive, negative, or bipolar EPSPs depending on the electrodes position in the CA1 area. Synchronization between the neuronal response and stimulus shows that the response is caused by the stimulation.

### Effect of stimulus amplitude on neuronal responses

**Fig 7:**
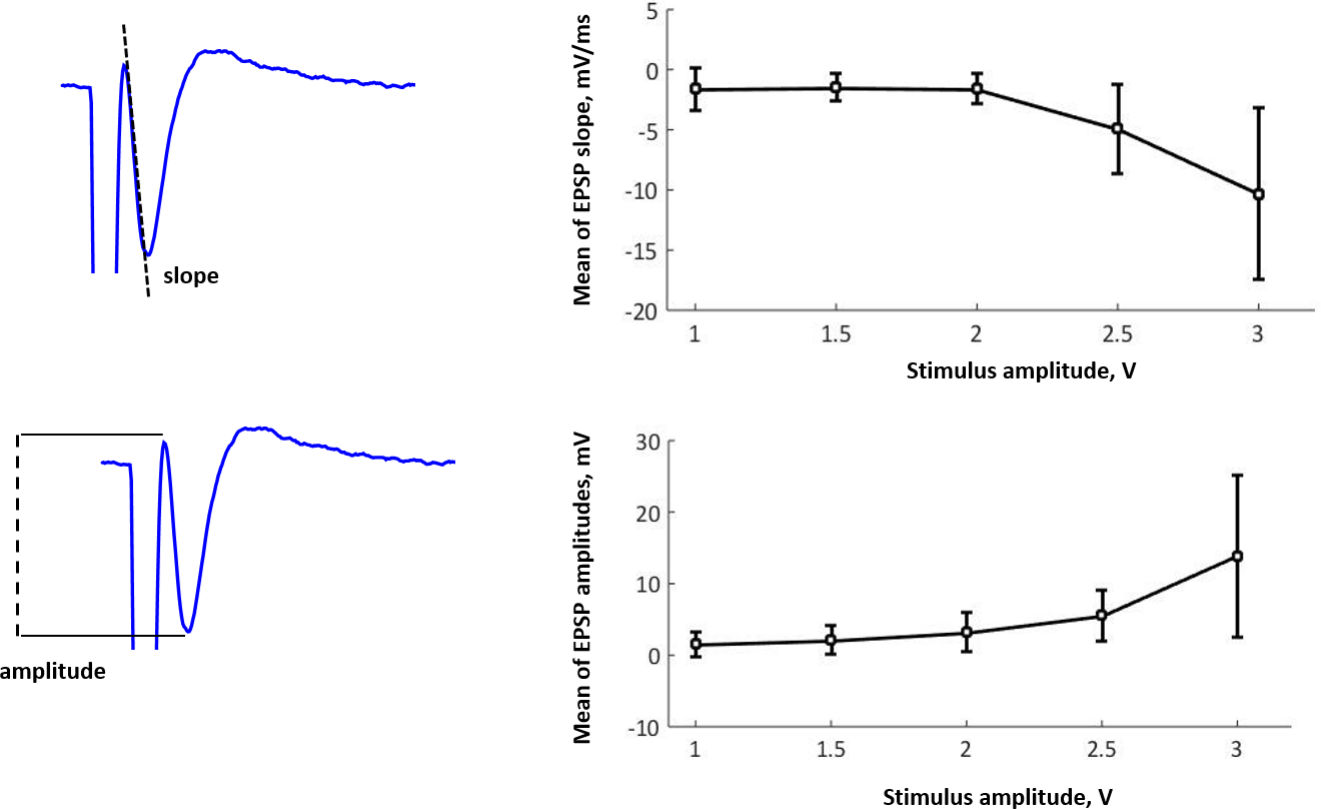
(a) Slope and (b) amplitude calculations from EPSP sample recordings of a single hippocampal neuron in CA1 area. (c,d) Mean of EPSP (c) slope and (d) amplitude during stimulation.

The population spikes of the CA1 hippocampal neurons are recorded after the Shaffer collateral stimulation. The dependences of the neuronal response on the stimulus amplitude are shown in Fig 7, where one can see that for small stimulus amplitudes (below 2 V) both the EPSP amplitude and the slope change slowly as the stimulus amplitude is increased. If the amplitudes were higher than 3 V we would not be able to see the correct responses because the neurons would die under such stimulation.

The EPSP slope characterizes the potential slowing rate. It is a more precise indicator of electrophysiological activity of hippocampal pyramidal neurons than the amplitude, because under strong stimulation, the excitatory postsynaptic potentials are noisy due to population spikes, and therefore the response amplitude cannot be correctly measured.

Our results demonstrate a biological plausibility in controlling both the EPSP slope and the amplitude by changing the amplitude of stimuli from the hybrid neural circuit. This reveals that the developed hybrid neural circuit can act as an adequate and effective artificial excitatory “cell” in the hippocampal circuit and control synaptic transmission in the hippocampus.

## Conclusions

In this paper, we have proposed an optoelectronic system for stimulation of living neurons, consisted of an electronic neuron generator, an optical fiber, and a photoelectrical converter. We have implemented this system for electrical stimulation of hippocampal living neurons. The main advantage of our system over other similar stimulators is that instead of metallic wires we used optical fiber signal transmission from the electronic neuron to living neurons. Due to galvanic isolation in the optical fiber, possible electrical disturbances and breakdowns are avoided. Although optical isolation (optocoupler or photocoupler) is quite common in electronics, the use of the optical fiber has another important advantage. Namely, the efficiency of the proposed device in manipulating neural dynamics can be further increased by implementing an active optical fiber (“optical synapse”) instead of the passive one [27,28]. This will allow adaptive connection where the transfer efficiency depends on the fiber gain factor, so that the doped fiber could evoke synaptic plasticity that opens new prospects in neuroprosthetics.

The proposed optoelectronic system (hybrid neural circuit) is very effective for stimulating electrophysiological living neurons in the hippocampus and can be used in restoring brain activity/individual regions or replacing individual parts of the brain (neuroprosthetics) which were damaged due to a trauma or neurodegenerative diseases.
